# Perinatal depression and omega-3 fatty acids: A Mendelian randomisation study

**DOI:** 10.1016/j.jad.2014.04.077

**Published:** 2014-09

**Authors:** Hannah Sallis, Colin Steer, Lavinia Paternoster, George Davey Smith, Jonathan Evans

**Affiliations:** a MRC Integrative Epidemiology Unit, School of Social and Community Medicine, University of Bristol, Barley House Oakfield Grove, Bristol BS8 2BN, UK; b Centre for Academic Mental Health, School of Social and Community Medicine University of Bristol, Barley House Oakfield Grove, Bristol BS8 2BN, UK; c Centre for Child and Adolescent Health, School of Social and Community Medicine, University of Bristol, Barley House Oakfield Grove, Bristol BS8 2BN, UK

**Keywords:** Depression, Perinatal, Mendelian randomisation, Omega-3, ALSPAC

## Abstract

**Background:**

There have been numerous studies investigating the association between omega-3 fatty acids (FAs) and depression, with mixed findings. We propose an approach which is largely free from issues such as confounding or reverse causality, to investigate this relationship using observational data from a pregnancy cohort.

**Methods:**

The Avon Longitudinal Study of Parents and Children (ALSPAC) cohort collected information on FA levels from antenatal blood samples and depressive symptoms at several time points during pregnancy and the postnatal period. Conventional epidemiological analyses were used in addition to a Mendelian randomisation (MR) approach to investigate the association between levels of two omega-3 FAs (docosahexaenoic acid (DHA) and eicosapentaenoic acid (EPA)) and perinatal onset depression, antenatal depression (AND) and postnatal depression (PND).

**Results:**

Weak evidence of a positive association with both EPA (OR=1.07; 95% CI: 0.99–1.15) and DHA (OR=1.08; 95% CI: 0.98–1.19) with perinatal onset depression was found using a multivariable logistic regression adjusting for social class and maternal age. However, the strength of association was found to attenuate when using an MR analysis to investigate DHA.

**Limitations:**

Pleiotropy is a potential limitation in MR analyses; we assume that the genetic variants included in the instrumental variable are associated only with our trait of interest (FAs) and thus cannot influence the outcome via any other pathway.

**Conclusions:**

We found weak evidence of a positive association between omega-3 FAs and perinatal onset depression. However, without confirmation from the MR analysis, we are unable to draw conclusions regarding causality.

## 1 Introduction

Several studies have investigated the association between omega-3 fatty acids (FAs) and depressive disorders, with mixed results. Although it has long been established that docosahexaenoic acid (DHA), a member of the omega-3 family, can affect brain function and behaviour (Simopoulos, 2009), the precise relationship with depression is unknown. Evidence from several observational studies shows an inverse relationship between fish consumption and depression which would appear to lend support to this hypothesis, as fish is a major source of omega-3 FAs (Hibbeln, 1998, 2002; Tanskanen et al., 2001; Silvers and Scott, 2002). However, observational studies are subject to issues such as confounding and reverse causation, and intervention trials attempting to establish whether depression can be treated with omega-3 FAs have met with mixed success (Freeman et al., 2006; Rondanelli et al., 2011; Su et al., 2008; Makrides et al., 2010; Carney et al., 2009).

It has been suggested that the lack of association in some intervention studies could be due to the investigation of the wrong FAs, or even the wrong ratios and dosages of FAs included in the interventions (Martins, 2009). Alternative criticisms focus on the broad inclusion criteria around diagnosis and the range of scales used to measure depression, both of which can lead to a heterogeneous mix of cases (Martins et al., 2012; Mischoulon, 2011). Meta-analyses of these intervention studies fail to reach a consensus on the association between omega-3 FAs and depression (Appleton et al., 2008, 2010; Bloch and Hannestad, 2012; Martins, 2009; Martins et al., 2012), and difficulties arise when attempting to combine studies due to heterogeneity among the range of study designs, types and doses of FAs investigated. Alternative methods of investigating the relationship between FAs and depression have not, as of yet, been explored.

It is possible that any association between FA levels and depressive symptoms are absent other than in challenging conditions, such as during pregnancy. Due to the increased nutritional demands on the body and the suspected resultant decrease in levels of highly unsaturated brain FAs during the antenatal and postnatal periods, women may be especially at risk of developing depressive symptoms at this time (Su et al., 2008; Kendall-Tackett, 2010). The potential range of symptoms and diagnostic criteria used when assessing depression can result in a heterogeneous mix of cases with varying symptoms, which can make discovering associations problematic and findings difficult to generalise. Perinatal onset depression can be thought of as a more homogenous subgroup of depression cases. In addition to sex, these cases are likely to be more similar in terms of age and other characteristics, as well as all undergoing a similar stress event (i.e. labour). In this situation, we should have greater power to detect an association between depression and levels of FAs, assuming such a relationship exists.

The omega-3 family of FAs are thought to be particularly relevant to depression as these are involved in brain development (McNamara and Carlson, 2006; Stahl et al., 2008). In particular, reduced levels of DHA are associated with impairments in both cognitive and behavioural performances (Innis, 2007). Alpha-linolenic acid (ALA) and linoleic acid (LA), from the omega-3 and omega 6 families respectively, are described as essential FAs because the body lacks the enzymes to synthesise them and so both must be obtained through diet. Due to the inefficient conversion of ALA to the longer chain omega-3 FAs, such as DHA, an adequate supply of dietary omega-3 FAs is important, however the modern Western diet tends to consist predominantly of omega-6 FAs. DHA and arachidonic acid (AA), an omega-6 FA, are known to be vital during pregnancy for foetal brain development, as well as during lactation and throughout the life cycle (Simopoulos, 2009). During the last trimester, the foetal brain undergoes rapid growth and requires increased levels of DHA. As such, maternal FA status is likely to deteriorate while the foetal demand for DHA is high (Steer et al., 2012; Hornstra, 2000; Kendall-Tackett, 2010). Eicosapentaenoic acid (EPA) is a precursor to DHA, and beneficial effects in several conditions, such as schizophrenia and depression, have been linked to this FA (Emsley et al., 2002; Marshall and Rathbone, 2011). In studies investigating the association of omega-3 FAs and depression, randomised controlled trials have tended to focus on supplementation with EPA, or EPA administered in parallel with DHA (Appleton et al., 2010), making it difficult to tease apart the effect of either FA. Arguments in support of both EPA and DHA as the more beneficial FA have been put forward, with a meta-analysis by Martins (2009) concluding that EPA has a greater effect in alleviating depression. However, other studies have focused on DHA, given its role as the major FA constituent of brain phospholipids, and the fact that it is the predominant omega-3 FA obtained through fish consumption (Sinn et al., 2012; Meyer et al., 2013).

In this study we investigate the association between antenatal FA levels and perinatal depression using data from the Avon Longitudinal Study of Parents and Children (ALSPAC) cohort. ALSPAC is uniquely placed to investigate the causality of any association with data collected on both FA levels throughout pregnancy and depressive symptoms at several time points during pregnancy and the postnatal period. In this study, we use a Mendelian Randomisation (MR) approach within the analysis to investigate whether there is a causal link between levels of two omega-3 FAs (DHA and EPA) and perinatal depression.

**Fig. 1:**
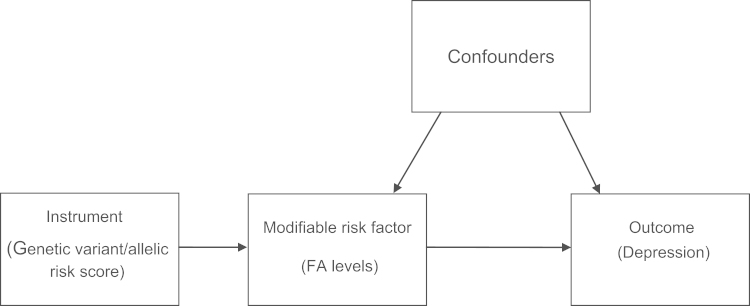
Directed acyclic graph illustrating Mendelian randomisation. In this model, allelic risk scores associated with FA levels are calculated and used to assess the association of both EPA and DHA with perinatal depression.

MR is a method of assessing causality from observational data through the use of instrumental variables. MR uses genetic variants, or scores constructed based on an individual׳s genotype across several variants, as a proxy for some modifiable risk factor associated with an outcome of interest, this process is illustrated in Fig. 1. The MR principle relies on both Mendel׳s first and second laws, which imply that genotypes transmit across conception to a viable conceptus, independent of both environment and other genetic variants (Davey Smith, 2011). Given these assumptions and, assuming the genetic variants are not associated with the outcome other than through the risk factor they act as a proxy for, we can make much stronger inferences about the causal nature of any association between the risk factor and the outcome (Davey Smith and Ebrahim, 2003). The effects given by the MR analysis should therefore be free from the problems of confounding and reverse causality to which observational epidemiology is prone. To our knowledge, this is the first study to investigate the association between FA levels and depression using an instrumental variable approach.

## 2 Methods

### 2.1 Sample

The ALSPAC study is a prospective cohort located in the South West of England. All women with an expected due date between April 1991 and December 1992 were eligible to join the study and 14,541 were initially recruited, resulting in 14,062 live births. Detailed information was collected on the mothers throughout pregnancy, and information has continued to be collected on the children, mothers and partners enroled in the study (Fraser et al., 2013; Boyd et al., 2013). Details of all available data are contained on the website through a fully searchable data dictionary (http://www.bris.ac.uk/alspac/researchers/data-access/data-dictionary/).

Ethical approval for the study was obtained from the ALSPAC Ethics and Law Committee and the Local Research Ethics Committees.

Women were included in this sample if data were available on genotype, FA levels and depressive symptoms during pregnancy or at 8 weeks postnatally. Mothers who lost a child during the neo-natal period (<28 days after childbirth) and those with a still birth were excluded from the analysis in order to avoid confounding with bereavement. Mothers with multiple births were also excluded from the analysis. In order to avoid problems arising through population stratification when using allelic risk scores, only women with a self-reported ethnicity of White European were included in the analysis. The total eligible sample size was therefore 3397 ALSPAC mothers.

### 2.2 Measures

#### 2.2.1 Edinburgh Postnatal Depression Scale

The Edinburgh Postnatal Depression Scale (EPDS) (Cox et al., 1987) was used to measure depressive symptoms at several time points during pregnancy and after childbirth. Although initially developed for use in the postnatal period, the EPDS has been validated for use both during pregnancy and following childbirth (Murray and Cox, 1990; Adouard et al., 2005). A threshold of >12 has been shown to highlight participants likely to be suffering from a depressive illness (Cox et al., 1987). This cut off is in accordance with that recommended by Cox et al. (1987).

Three sub-groups of depression were investigated; these were perinatal onset depression, antenatal depression (AND) and postnatal depression (PND). Women were defined as having a perinatal onset of depression if they were not depressed at 18 weeks gestation but went on to develop depressive symptoms at either 32 weeks gestation or 8 weeks postnatally. A score of >12 on the EPDS was used to define presence of depressive symptoms. PND and AND were defined according to EPDS scores at a single time point. Women were assigned PND case or control status according to responses given in questionnaires completed at approximately 8 weeks postnatally, those scoring >12 were classed as cases. AND was calculated by applying the same threshold to responses given at around 32 weeks gestation.

#### 2.2.2 Levels of docosahexaenoic acid and eicosapentaenoic acid

FA levels were extracted from antenatal blood samples collected from the mothers. Blood samples were taken at least once during pregnancy for FA composition analysis from 5222 mothers. The FA composition analysis has previously been described in detail by Newson et al. (2004). FA levels reported here refer to EPA or DHA as a proportion of the total FAs extracted from red blood cell (RBC) membrane phospholipids. The change in DHA levels refer to increments of 1%, while increments of 0.1% are used for EPA; for example a 3 unit change in DHA levels would correspond to a 3% change in DHA as a proportion of total RBC membrane FAs, while a 3 unit change in EPA would refer to a 0.3% change in EPA as a proportion of total RBC membrane FAs.

### 2.3 Genotype data

Genotyping was carried out at the Centre National de Génotypage (CNG) using the Illumina Human660W-quad array. Quality control measures included the removal of single nucleotide polymorphisms (SNPs) with >5% missing information, minor allele frequency (MAF) <1% and deviation from Hardy–Weinberg equilibrium (HWE) (*p*<1.0×10^−6^). Excluded individuals were those with >5% missing information, evidence of non-European ancestry from principal component analysis of the GWAS data or indeterminate X chromosome heterozygosity. A total of 8340 subjects and 526,688 SNPs passed these quality control filters.

### 2.4 Allelic risk scores

Allelic risk scores were calculated using results reported in Lemaitre et al. (2011), a GWAS of omega-3 FAs which did not include the ALSPAC sample. SNPs located on either chromosome 6 or 11 associated with *p*<5×10^−6^ were reported and where groups of SNPs were in high LD, the most significant of these was used to calculate the score, thus including only independent signals in the calculation. The ‘risk’ allele included in the score was that associated with a decrease in the relevant FA. Risk scores were calculated for each of DHA and EPA as a weighted sum of the number of risk alleles according to an individual׳s genotype across the relevant SNPs, and the effect sizes given by Lemaitre et al. (2011).

Locus zoom plots (Pruim et al., 2010) were used to visualise the LD structure between the SNPs reported in Lemaitre et al. (2011). Regions in LD (*r*^2^>0.7) were identified and the most significant SNP from each group was noted for use in the score (Supplementary Table 1).

### 2.5 Analysis

Analyses were carried out using Stata 12 (Statacorp, 2011). Initially the observational associations between perinatal onset depression and DHA and EPA were examined using logistic regression. Potential confounders were considered a priori and associations with depression and the FAs were investigated using chi-squared tests, *t*-tests and correlations (or a non-parametric equivalent). The potential confounding variables included maternal age, alcohol consumption during pregnancy, pre-pregnancy BMI, maternal social class and parity. Variables found to be significantly associated with both case status and FA level, or which were considered necessary to adjust for a priori, were considered for inclusion in the multivariable model.

The univariable models were restricted to mothers with complete data on all covariates included in the final multivariable model.

In a second step, 2 stage least squares regression (2SLS) was used in order to carry out the MR analyses. Allelic risk scores were calculated and used as instruments for EPA and DHA. The 2SLS method is generally intended for use with a continuous outcome. In the case of our binary depression outcomes, the results are still valid provided robust standard errors are used. Unlike the logistic regression which provides results in the form of odds ratios, the MR analysis presents results in terms of risk differences (RD). In order to make the MR estimates comparable to those calculated for the observational associations, risk differences were also calculated for these associations (Supplementary Table 2). A test of endogeneity was performed to test for differences between the estimates calculated using both the MR and conventional approaches.

The strength of the instrumental variables (IVs) used in the analysis was assessed using the Staiger-Stock rule of thumb in which instruments with an *F*-statistic of 10 or less are generally deemed to be weak instruments (Staiger and Stock, 1997).

### 2.6 Secondary analyses

Secondary analyses looked at the association between PND and AND with levels of EPA and DHA, using the same method of analysis described previously. Evidence of a non-linear relationship between FA levels and perinatal onset depression was also investigated. First, women in the lowest quartile of each FA were compared with the remaining women; in a further model, a quadratic term (the square of the FA level) was added into the model.

## 3 Results

### 3.1 Sample characteristics

The sample consisted of 306 mothers (11.5%) with a perinatal onset of depression, and 2357 controls who did not develop depressive symptoms either throughout pregnancy or by 8 weeks postnatally (Table 1). The number of mothers in the AND (prevalent antenatal cases) and PND (prevalent postnatal cases) subgroups were 441 (13.2%) and 265 (8.4%) respectively. The distribution of EPA was positively skewed, with 2 extreme observations among the controls exceeding 2%. These were excluded from the analysis as outliers, after which the mean value of EPA was 0.27% (SD=0.16). DHA showed less skew than EPA, and had a mean value of 2.31% (SD=1.33).

Among our sample, the mean maternal age at delivery was 28.9 years (SD=4.5) and the majority of women were among the higher social classes with only 9.9% falling in the lower (IV/V) category. In our sample, 81.8% of mothers breast fed at some point within the first 4 weeks of childbirth (Table 1).

Of the potential confounders investigated, there was evidence of an association between perinatal onset depression and both social class and maternal age at delivery (Table 2). FA levels showed an association with birth weight (EPA only), pre-pregnancy BMI, social class and maternal age at delivery (Table 3).

### 3.2 Allelic risk scores

The risk scores for both EPA and DHA were calculated from SNPs located on chromosomes 6 and 11 in the region of the FADS and ELOVL genes; the score for EPA was comprised of 23 SNPs while the score for DHA included 4 SNPs (for details, see Supplementary Table 1). Although the correlation was small, there was evidence of an association between DHA levels and the corresponding risk score (*r*=0.071, *p*=0.002), however there was very weak evidence of association between EPA levels and the allelic risk score (*r*=0.016, *p*=0.402). As a result, IV analysis of EPA would be subject to extreme weak instrument bias and as such we only report results of the IV analysis performed for DHA.

### 3.3 Perinatal onset depression

An unadjusted logistic regression of perinatal onset depression on EPA found no strong evidence of an association in our sample (*n*=2377, OR=1.05 [0.98,1.13]), the same also occurred when looking at DHA (*n*=2378, OR=1.06 [0.97,1.16]). After adjusting for social class and maternal age, the associations increased marginally, and showed weak evidence of an association with both EPA (OR=1.07 [0.99,1.15]) and DHA (OR=1.08 [0.98,1.19]) (Table 4).

Using the 2SLS approach, the association with DHA attenuated and there was no longer evidence of an association when adjusting for social class and maternal class (RD=0.08 [−0.05,0.21]) (Table 5). The estimates calculated in the MR and the observational analysis were not found to differ when performing a test of endogeneity (*p*=0.230). An assessment of instrument strength indicates that the IV used for DHA was of an acceptable level (*F*=12.01).

### 3.4 Antenatal depression

Unadjusted logistic regression showed no association between either EPA (OR=0.95 [0.89,1.02]) or DHA (OR=0.96 [0.88,1.05]) with AND. A model adjusting for social class and maternal age again found no strong evidence of an association with either of the FAs and AND (EPA: OR=0.97 [0.91,1.04], DHA: OR=0.99 [0.91,1.07]) (Table 4).

The MR analysis of DHA shows a similar trend (unadjusted: RD=0.07 [−0.08,0.22], adjusted: RD=0.05 [−0.09,0.19]) and found no robust evidence of an association with AND (Table 5). Once again a test of endogeneity found no substantial difference between the estimates from the MR and the observational analyses (*p*=0.440). The instrument for DHA remained of an acceptable strength (*F*=11.97).

### 3.5 Postnatal depression

A logistic regression of PND on EPA showed no substantial evidence of association either in an unadjusted model or when adjusting for social class and maternal age (unadjusted OR=1.04 [0.96,1.12]; adjusted OR=1.04 [0.96,1.13]). This pattern remained when analysing the association between PND and DHA (unadjusted OR=1.03 [0.95,1.12]; adjusted OR=1.03 [0.93,1.14]) (Table 4).

Again, the MR analysis found no robust evidence of an association between DHA and PND (unadjusted RD=0.03 [−0.08,0.14]; adjusted RD=0.02 [−0.08,0.13]) (Table 5). As in the previous analyses, the estimates from the MR and the observational analysis were not found to differ (*p*=0.700). The strength of the DHA IV remained a suitable strength for use in the analysis (*F*=14.53).

### 3.6 Non-linearity

There was no robust evidence of a non-linear association between levels of either FA and depression. This remained the case when social class and maternal age at delivery were adjusted for.

## 4 Discussion

When using logistic regression, a conventional observational approach to the analysis, we found weak evidence of an association between both EPA and DHA with perinatal onset depression. By including only women who were without depressive symptoms at the beginning of pregnancy, we have selected those who developed depression during a time when FA status is likely to be depleted - the potentially ‘pure’ cases of depression onset due to pregnancy. Mothers without depressive symptoms at the start of their pregnancy may have been initially less vulnerable to depression, and the change in FA status could have been the trigger for their development of depressive symptoms. If omega-3 FAs are associated with increased vulnerability to depression, then it seems plausible that this association would be more apparent among women who were otherwise at a lower risk for the disorder. The results of the analysis suggest that higher, rather than lower, levels of FAs are associated with an increased vulnerability to perinatal onset depression. The analysis provided weak evidence that a 0.1% increase in EPA was associated with a 6% increased odds of a perinatal onset, while a 1% increase in DHA was associated with an 8% increase in odds of perinatal onset depression. This is in contrast to our initial expectation that a reduction in FAs could lead to an increase in depressive symptoms.

When investigating women with a perinatal onset, the sample size is around 2378. The analysis of AND and PND, regardless of the timing of onset, gives us a slightly larger sample size (*n*=2912 and 2757), however among these groups we found no evidence of association. The combination of perinatal onset cases with women who were depressed throughout, thus including those in whom the onset was unrelated to pregnancy, could go some way to explaining the lack of association found among the AND and PND groups. Although the MR analysis shows no robust association even among the subset of women with a perinatal onset, much larger sample sizes are required for this approach to the analysis to be adequately powered. As a result, it is perhaps unsurprising that no association was found using the 2SLS method. For both the AND and PND groups, the MR analysis confirms the results shown in the simple logistic regression, in that there was no association found between either of the omega-3 FAs and depression. However, this may give us more confidence that there is no strong association present in the dataset that is being masked by confounding.

Although many studies have attempted to unpick the association between FA levels and depression before, to our knowledge this is the first to do so using an MR approach. ALSPAC is a unique resource in that it has available data on blood FA levels, genotyping data and information on depression at multiple times point during the perinatal period. Previous studies have differed greatly in the combination of FAs investigated, the doses and methods of intervention, which makes them difficult to combine in a pooled analysis. Here, we attempted to investigate causality using observational data available from a large sample of pregnant women. In order to have adequate power to detect an effect, should one be present, the MR analysis would require replication in a much larger sample.

### 4.1 Strengths and limitations

A main strength of our study is that the ALSPAC study is a large prospective cohort collecting detailed information on a variety of areas; in addition to this we also have genetic data and biomarkers available on a large subset of these women. In this study our total eligible sample size was 3397 women, a relatively large sample with which to investigate the association of omega-3 FAs and perinatal depression.

A further strength of the analysis is that we have data available on the levels of both FAs we are interested in, rather than having to rely on a proxy measure, such as supplements or self-reported food frequency questionnaires. Additionally, the FAs used in the analysis were RBC phospholipids, which are a better measure of longer term dietary habits than plasma phospholipids, which are sensitive to recent intake (Sun et al., 2007). RBC phospholipids reflect dietary intake over the previous months, whereas plasma phospholipids would measure intake in the previous days or weeks. Given that this measure is more stable, our allelic risk score is therefore likely to be a better instrument for this than it would for a more frequently fluctuating measure given that non-fasting blood samples were used.

Another additional strength of this analysis is that the MR approach enables us to investigate associations free from issues of confounding and reverse causation, which are so often problematic in observational epidemiology. Under the MR assumptions, application of this technique can strengthen our confidence in conclusions when making causal inferences.

There are also several limitations with the current study which warrant discussion. Firstly, pleiotropy is always a potential problem when using genetic variants as IVs, when using an allele score this problem is magnified as there are more variants that could be pleiotropic. Pleiotropy occurs when a SNP is associated with several traits, rather than just the trait of interest. If this is the case, then the assumptions of the MR analysis could be violated if the risk score is associated with depression via traits other than the omega-3 FAs in question. In this case, it seems possible that the score could be associated with other FAs. The correlation between our FAs of interest, EPA and DHA, is fairly strong at 0.725 (*p*<0.001). Both FAs show signals in the regions of ELOVL2 on Chromosome 6 and the FADS gene cluster on Chromosome 11 (Lemaitre et al., 2011). This is also the case for docosapentaenoic acid (DPA), another member of the omega-3 family, while ALA, another omega-3 FA, and many of the omega-6 family are primarily associated with the FADS gene cluster. Thus it could be that any association we see is due to a FA other than those we have investigated, or even a combination of FAs.

The allele score constructed here for DHA is more specific to this FA than to other FAs on the omega-3 pathway, however, given that the FAs themselves are so interlinked, it is difficult to tease apart their effects and it is likely that any score will correlate, albeit to a lesser extent, with other omega-3 FAs. However, this is a general concern when looking at FAs rather than a limitation specific to this study.

Additionally, the time at which samples were taken could also be thought of as a limitation. Blood samples were taken throughout pregnancy; however there were higher levels of missing data at the beginning of pregnancy. In an attempt to make the FAs more comparable in terms of gestation, the measurement used in the analysis was the FA level extracted from the last blood sample taken. A sensitivity analysis controlling for gestation at which the FA measurement was collected showed a negligible difference between the results of the two models.

In addition to the limitations with our FA exposure variable, the depression outcome variable also presents some challenges. Psychiatric outcomes are notoriously difficult to define, cases can present with a whole range of symptoms, which can make associations harder to establish. In our sample, cases were chosen based on a self-reported scale and thresholds were used to distinguish mothers most likely to be suffering a depressive disorder, rather than being based on a clinical diagnosis. Although the EPDS has been widely validated and shown to perform well, using cases based on a clinical diagnosis could potentially have increased our power to detect any association present.

Finally, although our eligible sample size was large, the use of IV methods requires very large numbers in order to have power to detect an effect, and so our MR analysis may still be lacking in power. A crude estimate, based on the reduction in standard errors that would be necessary to detect an effect of the magnitude of our estimates, points to an increase in sample size of around 4 times the current number (Wooldridge, 2002). Although the IV for DHA was found to be of a suitable strength, the score calculated for EPA was too weak to act as an effective instrument for EPA levels in our sample.

## 5 Conclusion

In conclusion, we found a weak association between increased omega-3 FA levels and perinatal onset depression in our sample when adjusting for maternal age and social class. However, these associations attenuated when using an MR approach and so we are unable to make inferences surrounding causality. When looking at PND and AND, we found no robust association with either of the omega-3 FAs investigated.

## Role of funding source

The UK Medical Research Council and the Wellcome Trust (Grant ref.: 092731) and the University of Bristol provide core support for ALSPAC. HS was funded by a Wellcome Trust 4-year Ph.D. studentship in Molecular, Genetic and Life Course Epidemiology (WT099871MA). HS, LP and GDS work in a Unit that receives research funds from the MRC. The funders had no role in study design, data collection and analysis, decision to publish or preparation of the manuscript.

## Conflict of interest

All authors declare that they have no conflicts of interest.

## Figures and Tables

**Table 1:** Perinatal onset depression sample characteristics.

	PoD Cases^a^ (*n*=306)	Controls (*n*=2357)	Overall
EPA – mean % (SD)	0.28 (0.17)	0.27 (0.16)	0.27 (0.16)
DHA – mean % (SD)	2.38 (1.38)	2.30 (1.33)	2.31 (1.33)
Birth weight – mean kg (SD)^b^	3.43 (0.50)	3.47 (0.50)	3.46 (0.50)
Maternal age at delivery (SD)	28.2 (4.5)	29.0 (4.4)	28.9 (4.5)
Pre-pregnancy BMI (SD)^c^	23.0 (4.8)	22.8 (3.5)	22.9 (3.7)

Gender of child – *n* (%)			
Male	157 (51.3)	1156 (49.1)	1313 (49.3)
Female	149 (48.7)	1201 (50.9)	1350 (50.7)

Breast feeding^d^ – *n* (%)			
No	77 (25.8)	397 (17.2)	474 (18.2)
Yes	222 (74.2)	1914 (82.8)	2136 (81.8)

Social class^e^ – *n* (%)			
High (I/II)	84 (31.5)	864 (40.9)	948 (39.9)
Mid (III)	149 (55.8)	1045 (49.5)	1194 (50.2)
Low (IV/V)	34 (12.7)	202 (9.6)	236 (9.9)

Parity^f^ – *n* (%)			
0	135 (44.7)	1100 (47.1)	1235 (46.8)
1	105 (34.8)	837 (35.8)	942 (35.7)
2+	62 (20.5)	401 (17.1)	463 (17.5)

Alcohol consumption during pregnancy^g^ – *n* (%)			
0 units	144 (68.2)	1104 (66.5)	1248 (66.7)
1–4 units	40 (19.0)	388 (23.3)	428 (22.9)
5+ units	27 (12.8)	169 (10.2)	196 (10.5)

^a^PoD=Perinatal onset depression. Missing data: ^b^Birth weight – 29, ^c^Pre-pregnancy BMI – 193, ^d^Breast feeding – 53, ^e^Social class – 285, ^f^Parity – 23, ^g^Alcohol consumption – 791 (questions relating to alcohol consumption were included from 15/07/91).

**Table 2:** Univariable associations with perinatal onset depression.

**PoD**^a^	OR	95% CI	*p* value	*N*
Birth weight (kg)	0.88	0.69,1.11	0.275	2634
Maternal age at delivery	0.96	0.93,0.99	0.004	2663
Pre-pregnancy BMI	1.01	0.98,1.05	0.528	2470

Social class				
Mid (III)	1.47	1.11,1.94	0.008	2378
Low (IV/V)	1.73	1.13,2.65		

Parity				
1	1.02	0.78,1.34	0.357	2640
2+	1.26	0.91,1.74		

Alcohol consumption				
1–4 units	0.79	0.55,1.14	0.232	1872
5+ units	1.22	0.79,1.91		

a PoD=perinatal onset depression.

**Table 3:** Univariable associations with omega-3 FA levels (% total RBC phospholipid FAs) –PoD^1^ controls only.

FA			Beta	95% CI	*p* value	*N*
Birth weight	EPA		−0.18	−0.31,−0.05	0.008	2327
	DHA		−0.06	−0.17,0.04	0.245	2329
Alcohol consumption	EPA	1–4 units	0.07	−0.12,0.25	0.187	1660
		5+ units	0.24	−0.02, 0.50		
	DHA	1–4 units	0.13	−0.03,0.28	0.155	1661
		5+ units	0.15	−0.07,0.37		
Pre-pregnancy BMI	EPA		−0.03	−0.05, −0.01	0.004	2190
	DHA		−0.02	−0.04,−0.01	0.002	2192
Parity	EPA	1	−0.11	−0.26,0.03	0.321	2336
		2+	−0.06	−0.24,0.13		
	DHA	1	−0.13	−0.25,−0.01	0.112	2338
		2+	−0.03	−0.18,0.12		
Social class	EPA	Mid (III)	−0.149	−0.297,−0.002	<0.001	2110
		Low (IV/V)	−0.500	−0.750,−0.249		
	DHA	Mid (III)	−0.148	−0.27,−0.03	<0.001	2111
		Low (IV/V)	−0.44	−0.65,−0.24		
Maternal age at delivery	EPA		0.03	0.01,0.04	<0.001	2355
	DHA		0.02	0.01,0.03	<0.001	2357

1 PoD=perinatal onset depression.

**Table 4:** Logistic regression of depression on omega-3 FA levels (% total RBC phospholipid FAs).

	OR	95% CI	*p* value	*N*
**Unadjusted model**				
**EPA**				
Perinatal onset	1.05	0.98,1.13	0.168	2377
Antenatal depression	0.95	0.89,1.02	0.168	2911
Postnatal depression	1.03	0.95,1.12	0.417	2756

**DHA**				
Perinatal onset	1.06	0.97,1.16	0.204	2378
Antenatal depression	0.96	0.88,1.05	0.360	2912
Postnatal depression	1.03	0.93,1.14	0.570	2757

**Adjusted model**^a^				
**EPA**				
Perinatal onset	1.07	0.99,1.15	0.083	2377
Antenatal depression	0.97	0.91,1.04	0.439	2911
Postnatal depression	1.04	0.96,1.13	0.301	2756

**DHA**				
Perinatal onset	1.08	0.98,1.19	0.102	2378
Antenatal depression	0.99	0.91,1.07	0.752	2912
Postnatal depression	1.04	0.94,1.15	0.421	2757

a Adjusted for social class (I/II, III or IV/V) and maternal age.

**Table 5:** IV regression of depression on DHA levels (% total RBC phospholipid FAs).

	RD^a^	95% CI	*p* value	Endogeneity test – *p* value	*N*
**Unadjusted model**					
Perinatal depression	0.09	−0.05,0.23	0.204	0.202	2378
Antenatal depression	0.07	−0.08,0.22	0.377	0.328	2912
Postnatal depression	0.03	−0.08,0.14	0.608	0.630	2757

**Adjusted model**^b^					
Perinatal onset	0.08	−0.05,0.22	0.214	0.230	2378
Antenatal depression	0.05	−0.09,0.19	0.464	0.440	2912
Postnatal depression	0.02	−0.08,0.13	0.660	0.700	2757

a RD=Risk difference.

b Adjusted for social class (I/II, III or IV/V) and maternal age.
