# Supplementary material for: Perinatal depression and omega-3 fatty acids: A Mendelian randomisation study
Source: J Affect Disord. 2014 Sep;166(100):124–31. doi: 10.1016/j.jad.2014.04.077 (PMC4101243; doi:10.1016/j.jad.2014.04.077)
Supplement: Supplementary file 1 — Supplementary Material [file mmc1.pdf]

Supplementary Table 1. SNPs used to create instrumental variables for DHA and EPA

SNP	Effect allele	Effect size	Chromosome
**DHA**			
rs2236212	C	-0.1132	6
rs3798721	A	0.1127	6
rs3846851	A	-0.0821	6
rs174555	T	0.0758	11
**EPA**			
rs7773173	C	0.0245	6
rs9393915	T	0.0389	6
rs3798713	C	0.035	6
rs10897208	A	0.0599	11
rs11033114	A	-0.0947	11
rs1109748	A	-0.0535	11
rs174448	A	0.0536	11
rs174468	A	0.05	11
rs174469	T	-0.0573	11
rs174532	A	0.0574	11
rs174538	A	-0.0834	11
rs174570	T	-0.0813	11
rs17762402	A	0.0869	11
rs17764324	T	-0.0483	11
rs2521568	C	-0.04	11
rs2845573	A	0.093	11
rs412334	T	0.044	11
rs498793	T	0.0351	11
rs650436	T	0.029	11
rs7102974	T	-0.1382	11
rs7394871	A	-0.0912	11
rs740006	T	-0.0655	11
rs7480288	T	0.0411	11

Supplementary Table 2: Regression of depression on DHA levels (% of total RBC phospholipids FAs) on the risk difference scale

	Observational analysis			IV analysis		
	RD[1]	95% CI	P value	RD[1]	95% CI	P value
**Unadjusted Model**						
Perinatal onset	0.006	-0.003,0.016	0.204	0.09	-0.05,0.23	0.204
Antenatal depression	-0.004	-0.013,0.005	0.360	0.07	-0.08,0.22	0.377
Postnatal depression	0.002	-0.005,0.010	0.570	0.03	-0.08,0.14	0.608
**Adjusted Model[2]**						
Perinatal onset	0.008	-0.002,0.017	0.104	0.08	-0.05,0.22	0.214
Antenatal depression	-0.001	-0.010,0.007	0.758	0.05	-0.09,0.19	0.464
Postnatal depression	0.003	-0.004,0.011	0.426	0.02	-0.08,0.13	0.660

[1] RD = Risk difference

[2] Adjusted for social class (I/II, III or IV/V) and maternal age
